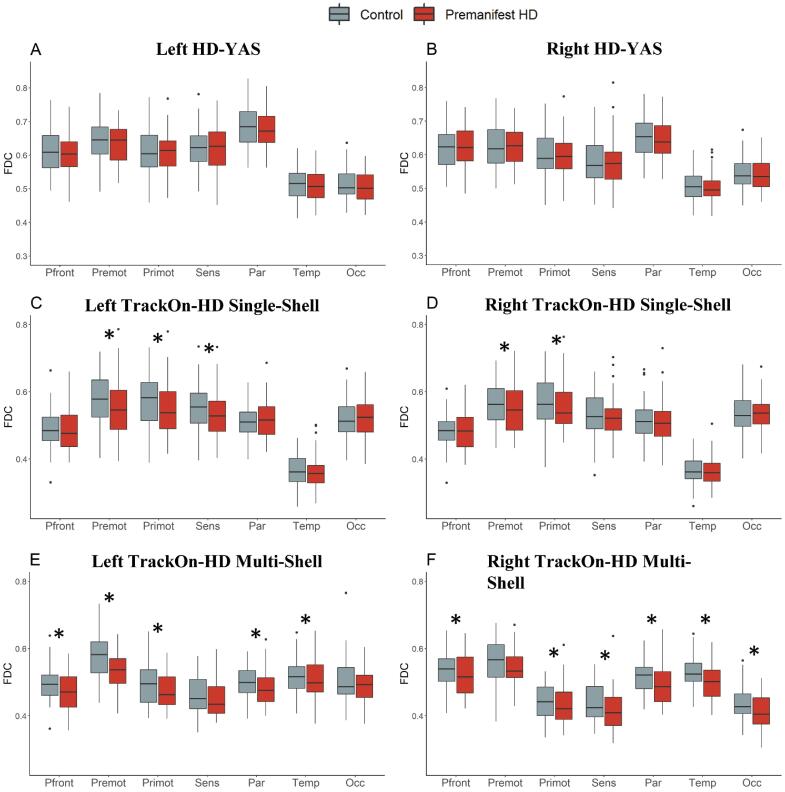# Timing of selective basal ganglia white matter loss in premanifest Huntington’s disease

**DOI:** 10.1016/j.nicl.2021.102927

**Published:** 2022-01-06

**Authors:** Paul Zeun, Peter McColgan, Thijs Dhollander, Sarah Gregory, Eileanoir B. Johnson, Marina Papoutsi, Akshay Nair, Rachael I. Scahill, Geraint Rees, Sarah J. Tabrizi, the TrackOn-HD and HD-YAS Investigators

**Affiliations:** a Huntington’s Disease Centre, Department of Neurodegenerative Disease, UCL Queen Square Institute of Neurology, University College London, WC1N 3BG, UK; b The Murdoch Children's Research Institute, Parkville Victoria 3052, Australia; c Max Planck UCL Centre for Computational Psychiatry and Ageing Research, UCL Queen Square Institute of Neurology, University College London, WC1N 3BG, UK; d UCL Institute of Cognitive Neuroscience, Queen Square, London WC1N 3BG, UK; e Dementia Research Institute at UCL, London WC1N 3BG, UK

**Keywords:** Huntington’s disease, Premanifest, Fixel-based analysis, Diffusion MRI, Striatum, Thalamus, CSD, Constrained spherical deconvolution, CSF, Cerebrospinal fluid, DTI, Diffusion tensor imaging, DCL, Diagnostic confidence level, dMRI, diffusion MRI, FC, Fibre cross-section, FD, Fibre density, FDC, Fibre density and cross section, FDR, False discovery rate, FOD, Fibre orientation distribution, HD, Huntington’s disease, HD-YAS, Huntington’s disease-young adult study, LMER, linear mixed effects regression, MRI, Magnetic resonance imaging, PreHD, Premanifest Huntington’s disease, TMS, Total motor score, UHDRS, Unified Huntington’s disease rating scale

## Abstract

**Objectives:**

To investigate the timeframe prior to symptom onset when cortico-basal ganglia white matter (white matter) loss begins in premanifest Huntington’s disease (preHD), and which striatal and thalamic sub-region white matter tracts are most vulnerable.

**Methods:**

We performed fixel-based analysis, which allows resolution of crossing white matter fibres at the voxel level, on diffusion tractography derived white matter tracts of striatal and thalamic sub-regions in two independent cohorts; TrackON-HD, which included 72 preHD (approx. 11 years before disease onset) and 85 controls imaged at three time points over two years; and the HD young adult study (HD-YAS), which included 54 preHD (approx. 25 years before disease onset) and 53 controls, imaged at one time point. Group differences in fibre density and cross section (FDC) were investigated.

**Results:**

We found no significant group differences in cortico-basal ganglia sub-region FDC in preHD gene carriers 25 years before onset. In gene carriers 11 years before onset, there were reductions in striatal (limbic and caudal motor) and thalamic (premotor, motor and sensory) FDC at baseline, with no significant change over 2 years. Caudal motor-striatal, pre-motor-thalamic, and primary motor-thalamic FDC at baseline, showed significant correlations with the Unified Huntington’s disease rating scale (UHDRS) total motor score (TMS). Limbic cortico-striatal FDC and apathy were also significantly correlated.

**Conclusions:**

Our findings suggest that limbic and motor white matter tracts to the striatum and thalamus are most susceptible to early degeneration in HD but that approximately 25 years from onset, these tracts appear preserved. These findings may have importance in determining the optimum time to initiate future disease modifying therapies in HD.

## Highlights

• Limbic and caudal motor cortico-striatal tracts appear to be selectively vulnerable to degeneration in the early premanifest Huntington’s disease (preHD), approximately 11 years from predicted onset.
• Similarly of cortico-thalamic connections, pre-motor and primary motor-thalamic connections appear selectively vulnerable to early degeneration.
• However these connections appear preserved in a preHD cohort 25 years from predicted onset.
• This highlights vulnerable subregions of the striatum and thalamus that may be important targets for future therapies that seek to prevent early neurodegeneration in preHD.

## 1 Introduction

Huntington’s disease (HD) is a neurodegenerative condition caused by a trinucleotide repeat expansion in the huntingtin gene. This results in degeneration of the cortico-basal ganglia white matter circuits resulting in progressive motor, cognitive and neuropsychiatric disturbance (McColgan and Tabrizi, 2018).

The basal-ganglia shows some of the earliest changes in premanifest (preHD), with loss of striatal grey matter grey matter (Tabrizi et al., 2011) and white matter (McColgan et al., 2015) occurring 15 years before disease onset. In manifest HD diffusion tractography (Bohanna et al., 2011, Marrakchi-Kacem et al., 2013) and anatomically based parcellations (Douaud et al., 2009) of striatal grey matter show group differences in cognitive and motor sub-regions. In preHD anterior caudate - frontal eye field connectivity has been associated with deficits in saccadic eye movements (Kloppel et al., 2008). A study from PREDICT-HD has investigated the white matter tracts of motor and sensory striatal sub-regions and demonstrated widespread group differences at baseline and change in the premotor striatum white matter over time (Shaffer et al., 2017).

Whilst converging literature indicates some of the earliest structural changes in preHD occur in the striatum and associated white matter, to date no study has investigated white matter tracts of all functional striatal and thalamic sub-regions in HD. Gene therapy trials in HD (Tabrizi et al., 2019) are targeting selective basal ganglia sub-regions, therefore understanding selective sub-region vulnerability is vital to designing these therapies.

Here we aim to address two questions: which striatal and thalamic sub-regions white matter tracts are most vulnerable in preHD and what is the time frame prior to symptom onset when these changes begin. In order to do this we performed diffusion MRI (dMRI) fixel-based analysis in two independent preHD cohorts; TrackON-HD (Klöppel et al., 2015) (approx. 15 yrs before disease onset) and the HD young adult study (HD-YAS) (Scahill et al., 2020) (approx. 25 years before disease onset).

## 2 Methods

### 2.1 Cohorts

**Fig. 1:**
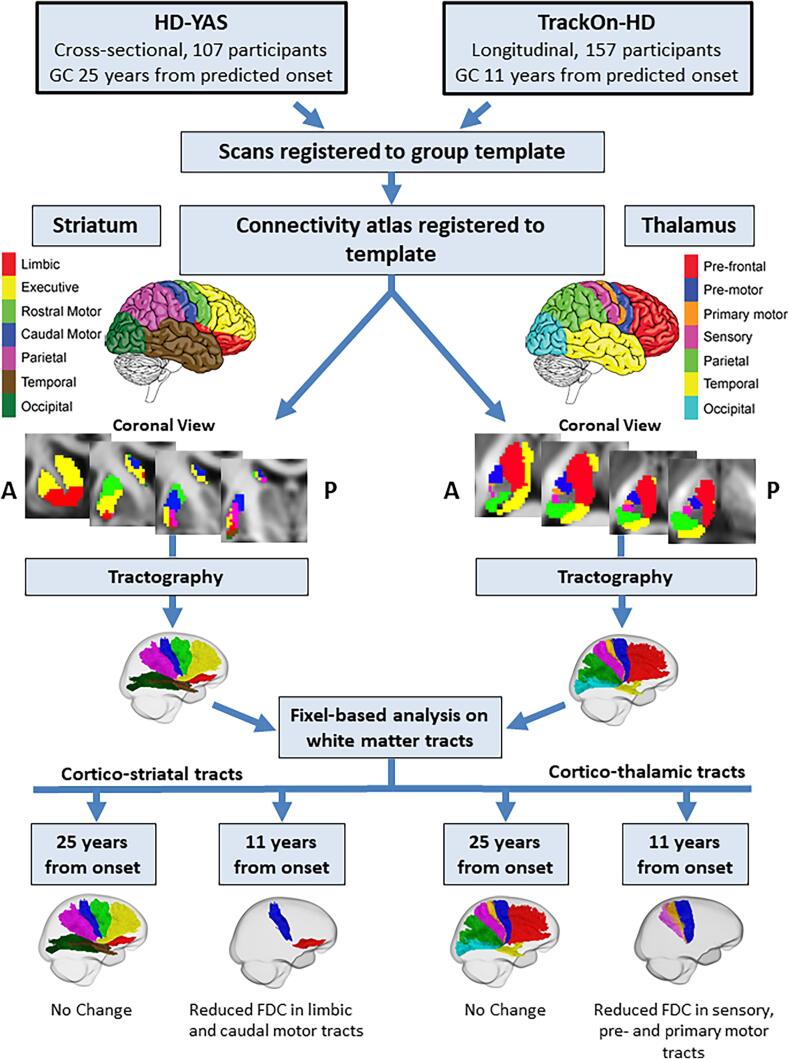
Overview of study methodology and key results. Diffusion data were analysed from two separate studies; HD-YAS, where HD gene carriers were 25 years before predicted disease onset, and TrackON-HD, where HD gene carriers were 11 years before predicted onset. For each study, scans were registered to a common template. Connectivity based atlases of the striatum and thalamus were registered to the group template. Diffusion tractography was performed on the group template to reconstruct each of the cortico-thalamic and cortico-striatal tracts in right and left hemispheres. Measures of FDC were then computed for each tract. In gene carriers 25 years before predicted onset, there were no differences in any cortico-striatal or cortico-thalamic tract. In HD gene carriers 11 years from predicted onset, we found reduced FDC in limbic and caudal motor cortico-striatal tracts and pre-motor, primary motor and sensory cortico-thalamic tracts cross-sectionally. There were no significant longitudinal changes in gene carriers 11 years before predicted onset. GC = Gene Carriers, FDC = Fibre density and cross-section.

An overview of study methodology and key results is provided in Fig. 1. For each cohort, gene carriers were required to have no diagnostic motor features of the disease (Unified Huntington’s disease rating scale (UHDRS) diagnostic confidence level (DCL) of < 4 and a CAG repeat size ≥ 40 where there is complete disease penetrance. The DCS is a measure of how confident a rater is in classifying an individual as manifest HD based on the UHDRS total motor score (TMS). The rating ranges from: 0 = normal to 4 = unequivocal signs of HD (>99% confidence). Typically scores of ≥ 15 on the TMS will trigger a DCS of 4 and hence diagnosis of manifest HD, although this depends on which items are scoring and the judgement of the rater. Controls were either gene negative (family history of HD but negative genetic test), partners of gene carriers, or members of the wider HD community (recruited through support groups or friends of participants). Participants were excluded if they were left-handed, ambidextrous, or had poor quality diffusion MRI data, as defined by visual quality control performed by PM and SG (Supplementary Table S1). Additional exclusion criteria included age below 18 or above 65, major psychiatric, neurological or medical disorder or a history of severe head injury. See Kloppel et al. (Klöppel et al., 2015) and Scahill et al. (Scahill et al., 2020) for detailed criteria. Participant demographics are summarised in Supplementary Table S2. HD-YAS preHD gene carriers were an average of 25 years before predicted disease onset, while Track-ON preHD gene carriers were an average of 11 years before disease onset. Year before predicted disease onset was calculated using the Langbehn equation (Langbehn et al., 2004). This is based on a parametric survival model developed using 2,913 HD gene carriers from 40 centres worldwide. Both studies were approved by the local ethics committee and all participants gave written informed consent according to the Declaration of Helsinki.

#### 2.1.1 HD-young adult study

To investigate how early basal ganglia white matter loss could be detected, we utilised MRI data from the HD-young adult study (HD-YAS) (Scahill et al., 2020). This was a single site cross-sectional study of gene carriers and controls aged 18–40. Data were collected between the August 2017 and April 2019. PreHD gene carriers required a disease burden score, a product of age and CAG repeat length (Penney et al., 1997) of ≤ 240 indicating these participants were ≥ 18 years before predicted disease onset. Mean TMS for both groups was 0 with a range of 0–5 in preHD and 0–1 in controls. Multi-shell dMRI data were analysed from 54 preHD and 53 control participants.

#### 2.1.2 TrackON-HD study

To investigate whether there is selective loss of specific basal ganglia white matter connections, we utilised MRI data from the TrackOn-HD study (Klöppel et al., 2015). This included single-shell diffusion data from preHD and control participants, scanned at 3 time-points one year apart over 2 years from 4 sites (London, Paris, Leiden, and Vancouver). Gene carriers were required to have a disease burden score of > 250 indicating participants were < 17 years before predicted onset. The total number of participants each year was as follows: year 1 (72 gene carriers, 85 controls), year 2 (81 gene carriers, 87 controls) and year 3 (80 gene carriers, 78 controls).

At the last TrackON-HD visit, participants at London and Paris sites had an additional multi-shell dMRI scan which included 33 gene carriers and 40 healthy control participants (Supplementary Table S2). The mean and range for TMS at the final timepoint in TrackON-HD was 5.9 (0–21) in preHD and 1.2 (0–7) in controls.

### 2.2 MRI acquisition

For the HD-YAS acquisition, dMRI data were acquired using a 3 T Siemens Prisma scanner, with a 64-channel head coil and b-values of 0, 300, 1000 and 2000 s/mm^2^ with 10, 8, 64 and 64-gradient directions respectively. Images had a voxel size of 2 × 2 × 2 mm^3^, 72 slices and a repetition time/time of echo of 3260/58 ms. One of the b = 0 volumes was acquired with reverse phase-encoding to allow for susceptibility-induced distortion correction. The scanning time was 10 min.

TrackON-HD single-shell dMRI were acquired using 4 different 3T scanners (Siemens TIM Trio MRI scanners at London and Paris, Phillips Achieva at Vancouver and Leiden) using similar imaging acquisitions across sites. Using a 12-channel head coil, dMRI were acquired with 42 unique gradient directions (*b* = 1000 s/mm^2^). Eight and one images with no diffusion weighting (*b* = 0) were acquired using the Siemens and Philips scanners, respectively. For the Siemens and Phillips scanners, repetition time/time of echo was 1300/88 and 1100/56 ms respectively. Voxel size for the Siemens scanners was 2 × 2 × 2 mm^3^, and voxel size for the Phillips scanners was 1.96 × 1.96 × 2 mm^3^. 75 slices were collected for each diffusion-weighted and non-diffusion-weighted volume with a scanning time of 10 min.

In additional to single shell dMRI, for the final time point in TrackON-HD, multi-shell dMRI data were also acquired at London and Paris, with b = 300, 700, 2000 sec/mm^2^ and 8, 32 and 64 gradient directions respectively; 14b = 0 images; voxel size = 2.5 × 2.5 × 2.5 mm^3^; repetition time/time of echo = 7000 ms/ 90.8 ms; 55 slices and a scanning time 15 min (Supplementary Table S2). Multi-shell and single shell acquisitions were analysed separated and study site was included as a covariate in all analyses.

### 2.3 Diffusion MRI processing

Preprocessing of dMRI was performed using MRtrix3 (Tournier et al., 2019) and FSL (Jenkinson et al., 2012) software packages. This included denoising of data (Veraart et al., 2016), Gibbs-ringing artefact removal (Kellner et al., 2016), eddy-current correction and motion correction (Andersson and Sotiropoulos, 2016), and up-sampling diffusion MRI spatial resolution in all 3 dimensions using cubic b-spline interpolation to 1.3 × 1.3 × 1.3 mm^3^ voxels (Dyrby et al., 2014). The upsampling of data helps to increase the anatomical contrast, which improves downstream spatial normalisation and statistics (Raffelt et al., 2017).

Three-tissue constrained spherical deconvolution (CSD) modelling of diffusion data was performed using MRtrix3Tissue (https://3Tissue.github.io), a fork of MRtrix3. For all data, response functions for single-fibre white matter as well as grey matter and CSF were estimated from the data themselves using an unsupervised method (Dhollander et al., 2016). For the single-shell TrackON-HD data, white matter-like fibre orientation distributions (FODs) as well as grey matter-like and CSF-like compartments in all voxels were then computed using Single-Shell 3-Tissue CSD (Dhollander and Connelly, 2016), whilst multi-shell multi-tissue CSD (Jeurissen et al., 2014) was utilised for the TrackON-HD multi-shell and HD-YAS data. Spatial correspondence was achieved by generating a group-specific population template with an iterative registration and averaging approach using white matter FOD images for 40 subjects (20 preHD and 20 controls, selected at random), in keeping with previous studies (Mito et al., 2018). Each subject’s FOD image was then registered to the template via a FOD-guided non-linear registration (Raffelt et al., 2011). For longitudinal data, an intra-subject template was produced using scans from all 3-time points before creating a common population template using the same registration approach.

### 2.4 Fixel based analysis

We utilised a fixel-based analysis to interrogate changes in white matter for this analysis. This was implemented in MRtrix3 (Tournier et al., 2019) and the methodology has been described previously (Raffelt et al., 2017). In brief, fibre density (FD) is a measure of the intra-axonal volume of white matter axons aligned in a particular direction (Raffelt et al., 2012). The fibre bundle cross section (FC) measures the cross-section of a fibre bundle by using the non-linear warps required to spatially normalise the subject image to the template image. The fibre density and cross section (FDC) is calculated by multiplying FD and FC providing one metric sensitive to both fibre density and fibre cross-section. For the main analysis, we report FDC only as it can be expected that neurodegeneration will cause combined reductions in both fibre density and bundle atrophy, however specific FD and FC measures are reported in the supplementary tables.

The common implementation of fixel-based analysis is a whole-brain analysis which then requires correction for a very large number of comparisons at a cost of reduced power to detect subtle changes. Since a large literature in preHD already exists to suggest the earliest structural changes occur in the striatum and associated white matter (Estevez-Fraga et al., 2021), we sought to specifically look at cortico-striatal white matter tracts only whilst still leveraging the advantages of fixel-based analysis in terms of diffusion signal processing. Seed-based diffusion tractography was used to delineate tracts of interest for subsequent fixel-based analysis. This approach has recently been successfully utilised in Parkinson’s disease (Zarkali et al., 2021). Cortico-thalamic white matter tracts were also included given their close proximity to previously described white matter changes and the important role of the thalamus in motor-cognitive loops affected by HD.

### 2.5 Generating tracts for analysis

We reconstructed distinct cortico-striatal and cortico-thalamic tracts using diffusion tractography on a group template before comparing FDC for each tract between groups (Fig. 1). Atlases of the striatum and thalamus derived using diffusion tractography were used to segment each structure in to 7 sub-regions per hemisphere based on the dominant area of cortical connectivity for each sub-region. Although several parcellation methods for these structures exist, including some derived from functional MRI datasets, it was considered appropriate to use dMRI derived atlases given the aim was to interrogate white matter integrity using dMRI data. For the striatum, the 7 sub-regions included limbic, executive, rostral motor, caudal motor, parietal, temporal and occipital regions (Tziortzi et al., 2014). The limbic sub-region connects to the orbital gyri, gyrus rectus and ventral anterior cingulate. The executive tract connects to dorsal prefrontal cortex. The rostral motor area connects to the pre-supplementary motor cortex and the frontal eye field region. The caudal motor tract connects the post-commissural striatum to the primary motor cortex, whilst parietal, temporal and occipital sub-regions connect to respective cortices. For the thalamic segmentation, the seven sub-regions are defined as those connected to prefrontal, premotor, primary motor, sensory motor, parietal, temporal and occipital cortices (Behrens et al., 2003). Within this segmentation, medial and dorsal thalamus including the mediodorsal nucleus connects to prefrontal and temporal regions. The ventral posterior nucleus connects to sensory motor cortex. The ventral lateral and anterior nuclei connect to primary motor and premotor cortex. The lateral posterior nucleus and parts of the pulvinar connect to parietal cortex.

The striatal and thalamic atlases were registered to the population template using NiftyReg (Modat et al., 2010) using a linear registration. Registration for each subject was visually checked to ensure accurate registration. To generate a tract for each striatal and thalamic sub-region, a tractogram was generated using probabilistic tractography on the population template. 20,000 streamlines were generated for each individual tract. Streamlines were initiated in each sub-region, with all other sub-regions masked out to avoid streamlines traversing other sub-regions and creating large amounts of overlap between the tracts. The result was a single fibre bundle for each sub-region connecting to its respective main cortical region. Fixel-based metrics were then calculated for all fixels (analogous to voxels) across the entire tract and averaged to generate a single measure of FDC for each tract. While mild striatal and cortical grey matter atrophy occurs in preHD, we did not perform volume normalisation as this can over compensate leading to spurious results (McColgan et al., 2015).

### 2.6 Statistical analysis

All statistical analysis was performed in MATLAB R2018a. Statistical analysis of the single-time point HD-YAS and TrackON-HD multi-shell data involved permutation testing (10,000 permutations) with two-tailed t-tests to investigate group differences. Age, gender, study site and education were included as covariates.

For the longitudinal Track-On analysis, linear mixed effects regression (LMER) was used, as it provides unbiased estimates under the assumption that the missing data is ignorable. It also accounts for dependence due to repeated measures. This approach has been used previously, where it is described in detail (McColgan et al., 2017a). We addressed multiple comparisons by applying a false discovery rate (FDR) approach to each separate cohort analysis and considered an FDR estimate of ≤ 0.05 to be significant.

To investigate whether changes in FDC show a relationship with clinical measures that are known to strongly relate to the integrity of the given white matter tract, clinical correlations for preHD in the TrackON-HD (n = 72) were performed for the tracts showing baseline change in the TrackON-HD single-shell results. Apathy scores from the Baltimore Apathy and Irritability scale (Chatterjee et al., 2005) were selected for limbic cortico-striatal tracts given the well described relationship between degeneration or injury in these tracts and apathy across numerous conditions (Le Heron et al., 2018). The UHDRS (Huntington Study Group, 1996) TMS and DCL were selected for caudal motor cortico-striatal tracts and thalamic tracts to premotor and primary motor cortices. Correlations were performed using partial correlations with age, gender, site, education and CAG included as covariates.

### 2.7 Data availability

The data that support the findings of this study are available from the corresponding author upon reasonable request.

## 3 Results

### 3.1 No significant differences in cortico-striatal and cortico-thalamic connections 25 years before predicted onset in HD gene carriers

**Supplementary figure 1:**
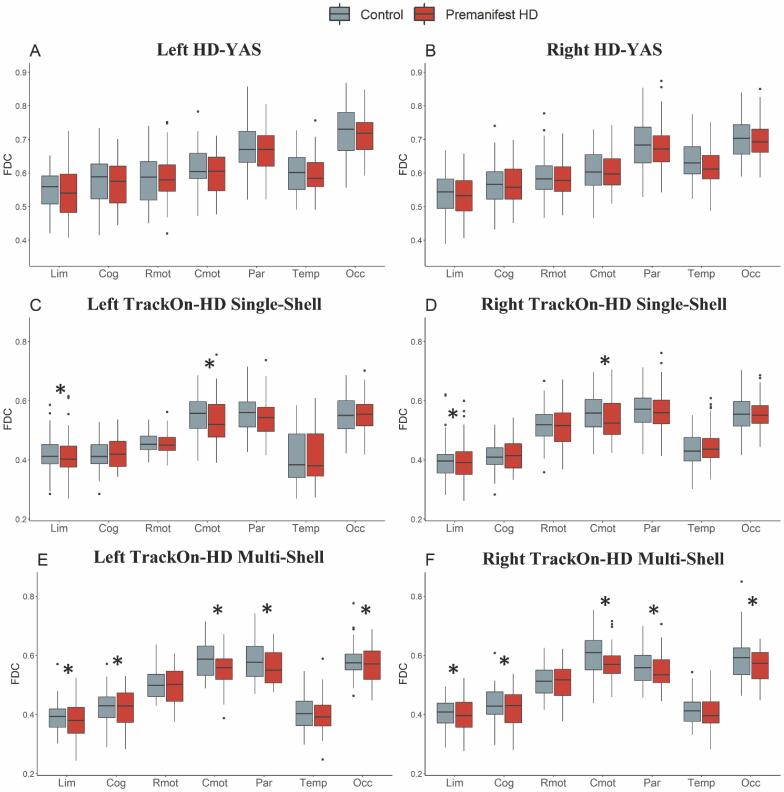


In the group of HD gene carriers approximately 25 years before predicted onset, no significant changes were seen in any cortico-striatal or cortico-thalamic tract compared to matched controls after FDR correction (Table 1, Table 2, Fig. S1 and 2A-B). This suggests that cortico-basal ganglia white matter connections 25 years before predicted disease onset are structurally preserved.

**Table 1:** Cortico-striatal fibre density and cross section (FDC) in HD-YAS.

Cortico-striatal Tract	Control Mean	PreHD Mean	SE	p	FDR
L Limbic	0.55	0.55	0.001	0.31	0.38
R Limbic	0.54	0.53	0.001	0.16	0.35
L Cognitive	0.58	0.57	0.001	0.32	0.38
R Cognitive	0.56	0.57	0.001	0.49	0.53
L Rostral Motor	0.58	0.59	0.001	0.54	0.54
R Rostral Motor	0.59	0.59	0.001	0.33	0.38
L Caudal Motor	0.62	0.60	0.001	0.04	0.35
R Caudal Motor	0.61	0.60	0.001	0.16	0.35
L Parietal	0.67	0.67	0.001	0.33	0.38
R Parietal	0.68	0.68	0.001	0.34	0.38
L Temporal	0.60	0.60	0.001	0.18	0.35
R Temporal	0.64	0.61	0.001	0.01	0.22
L Occipital	0.73	0.71	0.001	0.07	0.35
R Occipital	0.71	0.70	0.001	0.18	0.35

Unadjusted means displayed. γ = estimated group intercept difference, SE = standard error, p = p-value, FDR = false discovery rate corrected p-value.

**Table 2:** Cortico-thalamic fibre density and cross section (FDC) in HD-YAS.

Cortico-thalamic Tract	Control Mean	PreHD Mean	SE	p	FDR
L Prefrontal	0.61	0.61	0.001	0.18	0.35
R Prefrontal	0.62	0.62	0.001	0.32	0.38
L Premotor	0.65	0.63	0.001	0.08	0.35
R Premotor	0.63	0.62	0.001	0.30	0.35
L Primary Motor	0.61	0.61	0.001	0.34	0.38
R Primary Motor	0.60	0.60	0.001	0.34	0.38
L Sensory	0.62	0.62	0.001	0.52	0.54
R Sensory	0.58	0.58	0.001	0.33	0.38
L Parietal	0.68	0.67	0.001	0.09	0.35
R Parietal	0.65	0.64	0.001	0.17	0.35
L Temporal	0.52	0.51	0.0004	0.13	0.35
R Temporal	0.51	0.50	0.0004	0.05	0.35
L Occipital	0.51	0.50	0.0004	0.08	0.35
R Occipital	0.55	0.54	0.0004	0.18	0.35

Unadjusted means displayed. γ = estimated group intercept difference, SE = standard error, p = p-value, FDR = false discovery rate corrected p-value.

### 3.2 Anatomically specific basal ganglia white matter loss in preHD

**Fig. 2:**
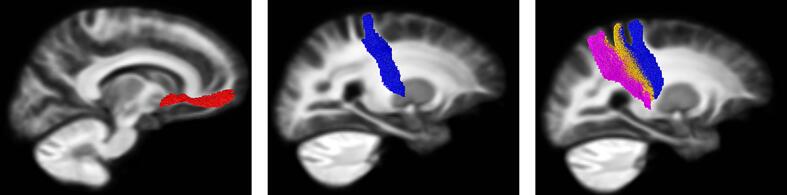
White matter tracts showing significant cross-sectional differences in the TrackON-HD cohort. White matter tracts that showed a significant (FDR < 0.05) group difference are shown on a single sagittal slice of the template tractogram. For cortico-striatal tracts these were limbic (red) and caudal motor (blue). For cortico-thalamic tracts these were premotor (blue), primary motor (orange) and sensory motor (purple). (For interpretation of the references to colour in this figure legend, the reader is referred to the web version of this article.)

We applied the same technique to a cohort of HD gene carriers closer to predicted onset to establish whether particular connections showed selective loss at this stage using the TrackON-HD single-shell cohort baseline results. For cortico-striatal tracts, significant reductions were seen bilaterally in limbic (left FDR = 0.002, right FDR = 0.02) and caudal motor (left FDR = 0.002, right FDR = 0.006) FDC (Fig. 2 and S1 C -D, Table 3). For cortico-thalamic tracts, significant reductions were seen bilaterally in pre-motor (left FDR = 0.005, right FDR = 0.02), primary motor (left FDR = 0.001, right FDR = 0.02) and left sensory (FDR = 0.006) FDC (Table 4, Fig. 2 and S2 C-D).

**Table 3:** Cortico-striatal fibre density and cross section (FDC) in TrackOn-HD Single-Shell Baseline.

Cortico-striatal Tract	Control Mean	PreHD Mean	γ	SE	p	FDR
L Limbic	0.42	0.41	−0.03	0.008	3.0 × 10^-4^	0.002
R Limbic	0.40	0.39	−0.02	0.007	0.005	0.02
L Cognitive	0.42	0.42	−0.01	0.006	0.05	0.08
R Cognitive	0.41	0.42	−0.01	0.006	0.13	0.15
L Rostral Motor	0.49	0.50	−0.007	0.008	0.38	0.55
R Rostral Motor	0.52	0.52	−0.02	0.008	0.04	0.09
L Caudal Motor	0.55	0.53	−0.03	0.008	4.9 × 10^-4^	0.002
R Caudal Motor	0.56	0.54	−0.03	0.008	0.001	0.006
L Parietal	0.55	0.55	−0.02	0.008	0.02	0.06
R Parietal	0.57	0.56	−0.02	0.008	0.07	0.11
L Temporal	0.41	0.41	2.2 × 10^-4^	0.006	0.97	0.97
R Temporal	0.43	0.44	0.001	0.006	0.84	0.84
L Occipital	0.55	0.55	−0.004	0.007	0.53	0.62
R Occipital	0.56	0.56	−0.01	0.007	0.08	0.11

Unadjusted means displayed. γ = estimated group intercept difference, SE = standard error, p = p-value, FDR = false discovery rate corrected p-value.

**Table 4:** Cortico-thalamic fibre density and cross section (FDC) in TrackOn-HD Single-Shell Baseline cohort.

Cortico-thalamic Tract	Control Mean	PreHD Mean	γ	SE	p	FDR
L Prefrontal	0.49	0.49	−0.02	0.007	0.03	0.05
R Prefrontal	0.48	0.48	−0.01	0.007	0.08	0.08
L Premotor	0.58	0.55	−0.03	0.009	0.002	0.005
R Premotor	0.51	0.51	−0.03	0.009	0.002	0.02
L Primary Motor	0.57	0.55	−0.03	0.008	1.30 × 10^-4^	0.001
R Primary Motor	0.57	0.55	−0.02	0.009	0.007	0.02
L Sensory	0.55	0.53	−0.02	0.008	0.003	0.006
R Sensory	0.53	0.52	−0.01	0.007	0.08	0.08
L Parietal	0.51	0.51	−0.006	0.006	0.35	0.40
R Parietal	0.51	0.51	−0.01	0.006	0.05	0.08
L Temporal	0.36	0.36	−0.007	0.005	0.15	0.21
R Temporal	0.36	0.36	−0.008	0.004	0.06	0.08
L Occipital	0.52	0.52	−0.002	0.007	0.08	0.35
R Occipital	0.54	0.53	−0.01	0.008	0.06	0.08

Unadjusted means displayed. γ = estimated group intercept difference, SE = standard error, p = p-value, FDR = false discovery rate corrected p-value.

We next investigated whether there were significant changes over a 2-year time period at this stage of the disease. No significant changes in any cortico-striatal or cortico-thalamic tracts were seen after FDR correction (Supplementary Table S3-4).

### 3.3 FDC changes using multi-shell acquisition at last time point in TrackOn-HD

We investigated the impact of dMRI acquisition on these results by repeating the analysis using the existence of an additional multi-shell dMRI scan, similar to the HD-YAS acquisition, in a subgroup of participants at the last time point in TrackON-HD. In this subgroup, we found widespread FDC changes in cortico-striatal and cortico-thalamic tracts (Fig. S1-2 E-F and Supplementary Table S5-6). Changes in FD and FC are presented in Supplementary Table S7-12.

### 3.4 Reductions in FDC correlate with a priori clinical measures

We assessed whether TrackON-HD baseline FDC changes were associated with relevant clinical measures in preHD (Supplementary Table S13). Mean and standard deviations for TMS were 5.9 ± 3.7 and 1.2 ± 1.5 for preHD and controls respectively from a total possible score of 124. For apathy, scores were 10.8 ± 7.4 and 8.3 ± 4.2 from a total score of 42. FDC in caudal motor-striatal tracts significantly correlated with UHDRS TMS (left r = -0.22, p = 0.007, right r = -0.21, p = 0.009) after adjustment for age, sex, site and education. There were also significant correlations between UHDRS TMS and pre-motor-thalamic FDC (left r = − 0.21, p = 0.01, right r = -0.20, p = 0.01) and primary motor-thalamic FDC (left r = -0.23, p = 0.004, right r = -0.20, p = 0.01). There were significant correlations between limbic cortico-striatal FDC and apathy (left r = 0.15, p = 0.07, right r = 0.22, p = 0.006).

## 4 Discussion

By studying two unique cohorts, we provide new insights into the time frame and anatomical specificity of basal ganglia white matter loss in preHD.

### 4.1 Selective vulnerability of striatal and thalamic cortical connections in preHD

The striatum and thalamus have a distinct topographical organisation of cortical white matter tracts forming individual subnetworks (Behrens et al., 2003, Tziortzi et al., 2014). By using detailed tractography-based striatum and thalamus connectivity atlases, we show that specific basal ganglia sub-region white matter tracts are more susceptible in preHD. Previous studies have investigated cortico-striatal white matter tracts to all cortical regions only in manifest disease, where widespread group differences are already apparent across numerous white matter tracts (Bohanna et al., 2011, Marrakchi-Kacem et al., 2013). The only previous study in preHD assessing specific white matter tracts of the striatum focused on motor, premotor and primary sensory white matter tracts only, reporting cross-sectional differences largely in the group < 8 years before predicted onset (Shaffer et al., 2017). In this study we apply fixel-based analysis, which is capable at resolving crossing white matter fibres at the voxel level (Raffelt et al., 2017). We present the first analyses to investigate all white matter tracts of striatal sub-regions in preHD and in doing so identify the caudal motor-striatal and limbic-striatal white matter tracts as being selectively vulnerable.

Compared to the striatum, the thalamus and its white matter tracts have been comparably lesser studied and are generally believed to be affected later in the disease course (Zeun et al., 2019). This study is the first to investigate thalamic sub-region white matter tracts in HD. We show that 11 years before disease onset there is selective vulnerability of pre-motor, primary motor and sensory thalamic white matter tracts. This is in keeping with post-mortem evidence of thalamic selective vulnerability, where selective degeneration of the motor ventrolateral nucleus and the centromedian nucleus, which helps integrate sensorimotor functions, has been observed (Rüb et al., 2016).

### 4.2 Changes in white matter tracts are associated with clinical measures

The clinical relevance of this basal-ganglia white matter tract selective vulnerability is demonstrated by negative correlations between the UHDRS TMS and FDC of the caudal motor-striatal, premotor-thalamic and primary motor-thalamic white matter tracts, such that lower FDC is associated with greater motor deficit. The association of thalamic white matter tracts with motor deficits suggests that in addition to the striatum, the degeneration of cortico-thalamic pathways is also likely to be significant in the emergence of the clinical motor manifestations of HD.

The significant positive correlation between limbic cortical-striatal FDC and levels of apathy was unexpected. However, apathy scores in this preHD cohort were low, did not correlate with disease burden and were similar to the control group. Hence this association should be interpreted with caution and may reflect the limited clinical utility of this clinical scale early in preHD. Nevertheless, converging literature across other neurodegenerative and cerebrovascular diseases associated with apathy has clearly demonstrated that degeneration or injury to this white matter pathway is associated with the emergence of apathy (Le Heron et al., 2018, Prange et al., 2019). It is therefore likely that the observed reduction in limbic cortico-striatal tract FDC is clinically significant and likely contributes to the emergence of apathy in HD later in the disease course.

### 4.3 White matter tracts appear preserved approximately 25 years from predicted onset

We show that cortico-striatal and cortico-thalamic white matter tracts appear structurally preserved in preHD approximately 25 years before clinical onset. To our knowledge, this is the first time that preserved white matter tracts across all striatal and thalamic sub-regions have been demonstrated in preHD. Recent evidence has suggested that the HD mutation may lead to abnormalities in striatal and cortical development (Barnat et al., 2020). Our findings indicate that there is no detectable developmental abnormality in the microstructure of cortico-striatal and cortico-thalamic white matter tracts in preHD, using novel fixel-based dMRI analysis, which has been shown to be more sensitive to neurodegeneration of white matter in Alzheimer’s disease (Mito et al., 2018) when compared to standard diffusion tensor imaging (DTI) approaches.

### 4.4 Emerging insights using fixel-based analysis in HD

This is the first application of a fixel-based analysis in preHD. Fixel-based analysis offers an improvement over previously studied DTI methods by accounting for crossing fibre populations to provide more reliable tractograms and account for the differing ways in which changes to intra-axonal volume may occur by quantifying both a measure of fibre density and a measure of fibre bundle cross section (Raffelt et al., 2017, Dhollander et al., 2021). Two studies have previously studied fixel-based analysis in manifest HD cohorts (Adanyeguh et al., 2021, Oh et al., 2021). Both reported reductions in fixel metrics in white matter pathways that include cortico-striatal and cortico-thalamic connections, namely the internal capsule and corticospinal tracts as well as the external capsule, corpus callosum and corona radiata. Both studies also found greater decreases in FC and FDC than FD, as reported in this study, reflecting more prominent atrophy of white matter tracts rather than reduction of fibre density. Similar to this study, Adanyeguh et al. also reported reductions in FC and FDC significantly correlated with the total motor score, reflecting the importance of these white matter tracts in the emergence of the motor manifestations of HD (Adanyeguh et al., 2021). The findings of this study extend the previous literature by showing that reductions in fixel metrics are also detectable in the premanifest period, but not in a cohort further from predicted onset, whilst highlighting the cortical-subcortical connections most affected early in preHD.

### 4.5 Improved signal-to-noise in fixel-based analysis with higher b-values

Previous studies have demonstrated an improved signal-to-noise in measuring FD at higher b-values than incorporated in this study, owing to improved suppression of the extra-axonal signal (Raffelt et al., 2017, Genc et al., 2020). To investigate the influence of different acquisition parameters, we replicated our analysis in a subset of the TrackON-HD cohort who had additional multi-shell dMRI at the final time point with b-values of up to 2,000 s/mm^2^. These results replicated the findings from the single-shell analyses and in addition revealed more widespread significant changes in FDC. This suggests improved sensitivity to white matter differences and further strengthens findings in the HD-YAS cohort with a similar acquisition, that cortico-basal ganglia white matter tracts are preserved. The apparent reduced sensitivity to change of lower b-value dMRI may also partly explain the lack of longitudinal changes seen in our TrackON-HD cohort whilst also likely reflecting the slow trajectory of white matter change in HD consistent with previous findings (Poudel et al., 2015).

### 4.6 Potential relevance of findings to therapeutics currently in development

Our findings suggest the initiation of disease modifying therapies very early in the premanifest period could help preserve these essential white matter tracts, which form the motor, cognitive and limbic cortico-basal ganglia circuitry. Among emerging approaches include viral-vector based therapeutics such as RNAi (Tabrizi et al., 2019). These must be injected directly into the brain and a significant mechanism of its distribution is via retrograde axonal transport (Evers et al., 2018). The striatum and thalamus, given their significant early involvement in HD and large spread of cortical connections are likely targets for injection in such therapeutics with the first such therapeutic with intra-striatal injection already in early clinical trials (Evers et al., 2018, Clinicaltrials.gov; NCT04, 2019). Therefore the results of this study suggest that initiating treatment early in the premanifest period may afford the best drug distribution for such therapeutics whilst white matter tracts remain preserved and highlight selectively vulnerable subregions that may represent optimal sites for injection to prevent early white matter degeneration in HD. The lack of longitudinal changes in this study reinforce results from previous literature in preHD that changes in diffusion measures occur slowly over time and as such are unlikely to be useful as biomarkers of disease progression (Zeun et al., 2019).

### 4.7 Limitations of current study

With respects to limitations of the current study, direct comparisons between these two cohorts are limited by differences in MRI acquisition, participant number, age and study design between the two cohorts that required different statistical methodology. However the acquisitions were very similar across sites and previous split site analyses of the Track-ON HD diffusion dataset has shown consistent findings across sites (McColgan et al., 2017a, McColgan et al., 2017b). In the Track-ON HD study there were significant age differences between preHD and controls, while this is an unavoidable consequence of the natural history of HD, we aimed to minimise this effect by including age as a covariate of no interest in all analyses.

The absence of significant difference between preHD and controls, 25 years before onset, does not exclude the possibility of subtle changes in cortico-basal ganglia white matter tracts or the possibility of functional changes, which could be measured using functional MRI or magentoencephalography, and this warrants further investigation. Longitudinal follow up of this cohort will also be important pinpoint the exact time when white matter loss begins.

## 5 Conclusion

These findings suggest that white matter tracts of cortico-basal ganglia functional sub-regions remain intact in preHD gene-carriers approximately 25 years before to predicted onset and that degeneration begins within an 11–25 year time frame from diagnosis. Selective vulnerability is seen in white matter tracts to the limbic and motor striatum and the motor and sensory thalamus. This indicates that initiation of disease modifying therapies before demonstrable changes have occurred could prevent neurodegeneration of these white matter tracts and highlights selectively vulnerable sub-regions of the striatum and thalamus that may be important targets for future therapies.

## CRediT authorship contribution statement

**Paul Zeun:** Conceptualization, Data curation, Formal analysis, Investigation, Methodology, Project administration, Writing – original draft. **Peter McColgan:** Conceptualization, Data curation, Formal analysis, Investigation, Methodology, Project administration, Writing – review & editing. **Thijs Dhollander:** . **Sarah Gregory:** Data curation, Writing – review & editing. **Eileanoir B. Johnson:** Data curation, Writing – review & editing. **Marina Papoutsi:** Data curation, Writing – review & editing. **Akshay Nair:** Data curation, Writing – review & editing. **Rachael I. Scahill:** Funding acquisition, Data curation, Writing – review & editing. **Geraint Rees:** Funding acquisition, Writing – review & editing, Supervision. **Sarah J. Tabrizi:** Funding acquisition, Writing – review & editing, Supervision.

## Declaration of Competing Interest

The authors declare that they have no known competing financial interests or personal relationships that could have appeared to influence the work reported in this paper.

## Figures and Tables

**Supplementary figure 2:**